# A case report of multiple primary malignant tumors: breast cancer and colorectal cancer

**DOI:** 10.3389/fonc.2025.1713186

**Published:** 2025-11-25

**Authors:** Mirinisa Kaicaier, Shengjie Liu, Yuhan Zhang, Xueping Hou, Yuying Wang, Aisaide Aikelaimu, Liyuan Ma, Weihua Jiang

**Affiliations:** The Second Department of Breast Surgery, The Affiliated Tumor Hospital of Xinjiang Medical University, Xinjiang Uygur Autonomous Region, Urumqi, China

**Keywords:** breast cancer, colorectal cancer, multiple primary malignant tumors, multidisciplinary team, chemotherapy, adverse reaction

## Abstract

Breast cancer and colorectal cancer are both common malignant tumors worldwide. This report describes a case of multiple primary malignant tumors (MPMT) in a 52-year-old female patient diagnosed successively with right breast triple-negative breast cancer (T2N1M0, stage IIb) and rectal moderately differentiated adenocarcinoma (pT3N0M0, stage IIa). Through a multidisciplinary team (MDT) approach, we developed a treatment strategy involving breast-conserving surgery for the breast cancer followed by laparoscopic radical resection for the rectal cancer. Postoperative sequential therapy comprised TP chemotherapy (albumin-bound paclitaxel plus carboplatin), radiotherapy, and capecitabine. The patient developed Grade IV bone marrow suppression during chemotherapy, which improved after aggressive supportive care. The patient is currently in disease remission. This case highlights the importance of multidisciplinary collaboration, genetic risk assessment, individualized treatment decisions, comprehensive tumor management, and robust supportive care in the diagnosis and treatment of multiple primary malignant tumors, providing valuable insights for managing similar clinical cases.

## 1 Introduction

Breast cancer exhibits unique epidemiological patterns and significant heterogeneity. It remains the most common malignancy among women globally and the second leading cause of cancer-related deaths worldwide (1). Its development is closely associated with multiple factors, including genetic susceptibility, and treatment strategies based on molecular subtypes are highly individualized (2–4). The disease demonstrates diversity in histological types, natural course, clinical manifestations, and treatment responses. Traditional breast cancer treatments include surgery, chemotherapy, radiation therapy, endocrine therapy, and targeted therapy (5). Based on current evidence, triple-negative breast cancer (TNBC) is best regarded as an umbrella term encompassing multiple distinct entities with significant genetic, transcriptional, histological, and clinical differences. Despite its highly aggressive clinical characteristics, multiple studies indicate that patients with TNBC are more likely to achieve pathological complete response following neoadjuvant chemotherapy (6–8). TNBC accounts for approximately 15% to 20% of all breast cancers (9). In recent years, the rise of precision medicine has ushered in a new era for breast cancer treatment, emphasizing the development of personalized therapeutic strategies based on the specific molecular characteristics of individual tumors (10). Furthermore, long-term management is crucial for patients with tumors such as breast cancer, as it directly impacts their quality of life and survival outcomes (11–13). Colorectal cancer is also a significant global health issue, ranking among the leading causes of cancer incidence and mortality worldwide (14). Its development is a complex, multi-step process involving the cumulative accumulation of mutations in multiple key genes, such as APC, KRAS, TP53, and SMAD4 (15–17). These mutations lead to the abnormal activation of signaling pathways including Wnt, EGFR/MAPK, and TGF-β, driving the sequential progression from normal mucosa to adenoma and ultimately to adenocarcinoma (18, 19). Although surgical resection remains the primary curative approach for early-stage colorectal cancer, preoperative neoadjuvant chemoradiotherapy for locally advanced rectal cancer, along with systemic chemotherapy, targeted therapy, and immunotherapy for advanced disease, have significantly improved patient outcomes (20, 21). Nevertheless, numerous challenges persist in the prevention and treatment of colorectal cancer, including treatment resistance due to tumor heterogeneity, poor response to immunotherapy in microsatellite stable (MSS) tumors, and the screening and management of genetically susceptible populations such as those with Lynch syndrome (22). Multiple-origin malignant tumors with ectopic sites are relatively uncommon in clinical practice. Their diagnosis and treatment strategies are more complex, requiring comprehensive consideration of the biological behavior, staging, and systemic condition of both tumors. Multidisciplinary team (MDT) collaboration plays a central role in the individualized management of such patients. We report a case of multiple primary ectopic carcinoma originating from breast and rectal cancer to explore its clinical management strategies.

## 2 Case data

A 52-year-old female patient was admitted on April 27, 2025, presenting with a right breast mass discovered one month prior. Physical examination: Both breasts are symmetrical. Skin shows no redness, swelling, ulceration, or orange-peel changes. A firm, poorly mobile mass measuring approximately 2×1 cm is palpable in the lateral aspect of the right breast, with no skin adhesion. No enlarged lymph nodes are palpable in either axilla or supraclavicular region.

**Figure 1:**
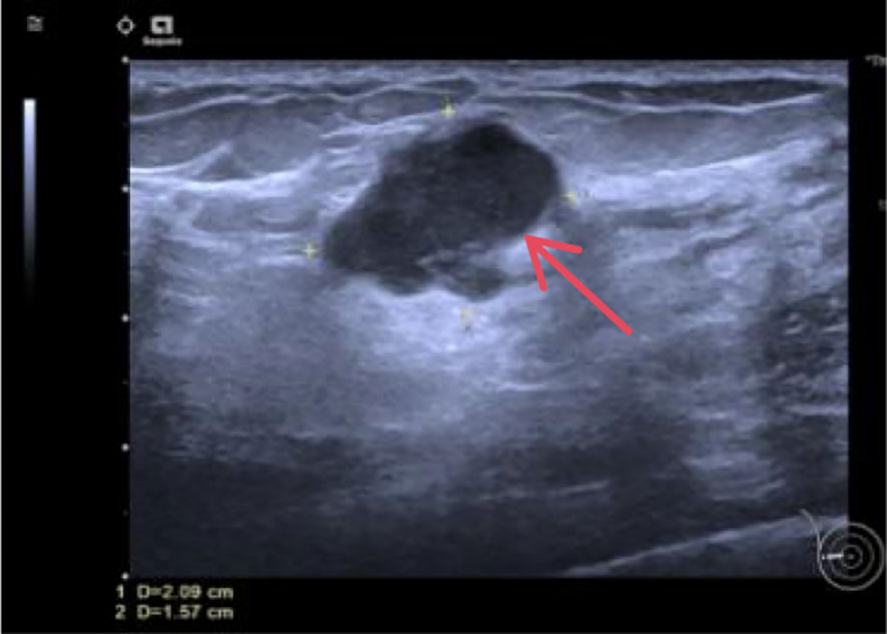
Color doppler ultrasound: A 2.1 x 1.6 x 1.6 cm irregularly shaped solid hypoechoic mass is noted at the 9 o’clock position in the right breast, approximately 3.7 cm from the nipple and 0.5 cm from the skin surface. The aspect ratio exceeds 1. The margins are ill-defined with a spiked appearance. Internal echoes are heterogeneous, showing multiple small punctate hyperechoic foci. Posterior acoustic attenuation is present. with a surrounding hyperechoic halo. CDFI: Rich blood flow is visible within the lesion. BI-RADS: Category 5 (the red arrow in the image points to the mass).

**Figure 2:**
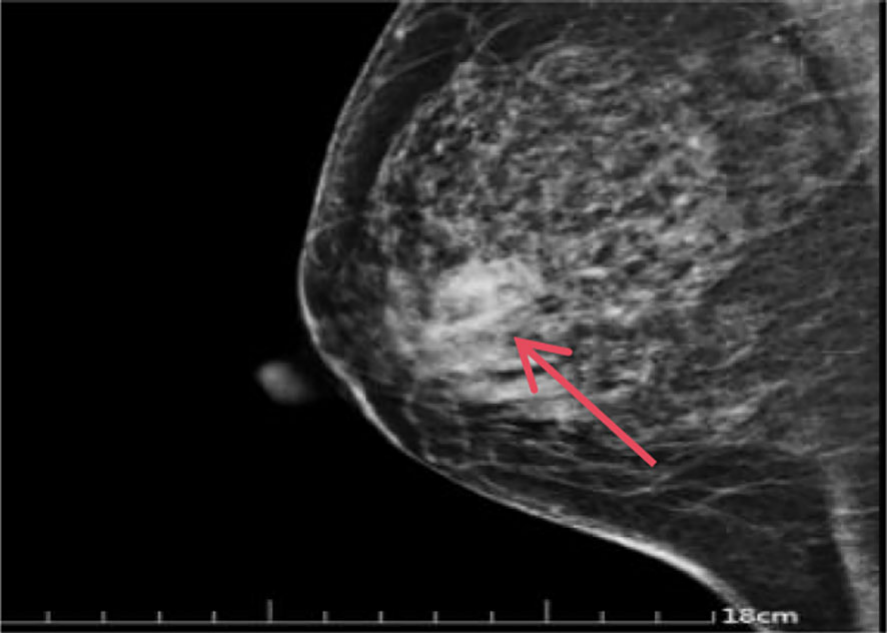
Mammography: A dense, irregular mass shadow is noted in the upper-mid outer quadrant of the right breast, measuring approximately 1.6 x 2.1 cm. The margins are irregular and lobulated, with adjacent structures disrupted. No significant nipple retraction is observed on the right side. Localized skin retraction with slight indentation is present. The corresponding subcutaneous fat layer appears cloudy with increased striation. BI-RADS: Category 4C (the red arrow in the image points to the mass.).

**Figure 3:**
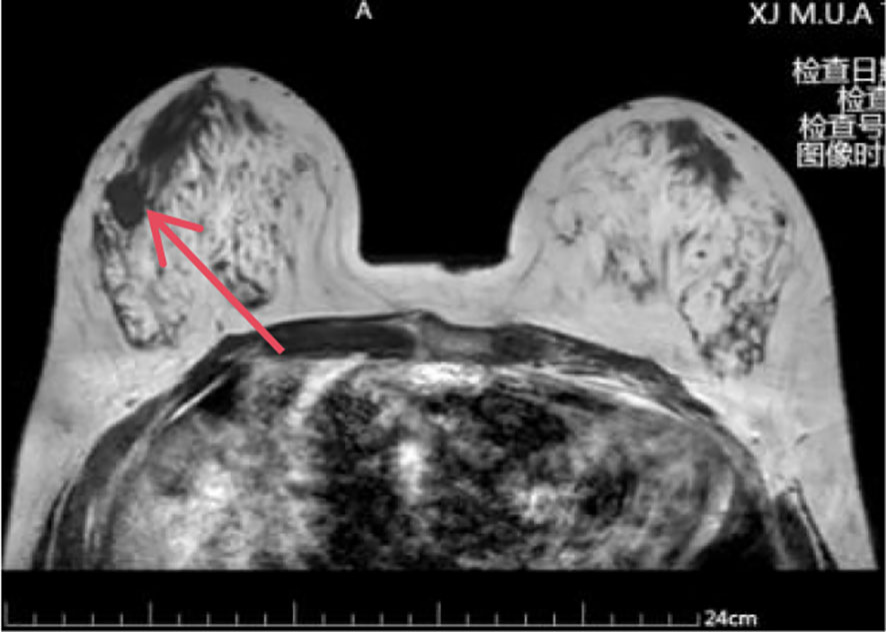
MRI: A round mass measuring approximately 1.9 × 1.4 × 1.7 cm is visible in the right breast’s outer lower quadrant.(The red arrow in the image points to the mass.).

Auxiliary Examinations: Breast Ultrasound: Reveals a solid mass in the right breast (BI-RADS: Category 5, Impression: Possible breast cancer) measuring approximately 2.1 x 1.6 x 1.6 cm (**Figure 1**); Mammogram: Suggests a right breast mass, suspected breast cancer [BI-RADS: Category 4C]. Size approximately 1.6 x 2.1 cm (**Figure 2**). Breast MRI: Suggestive of breast cancer in the right breast, lower outer quadrant, measuring approximately 1.9 x 1.4 x 1.7 cm (**Figure 3**). Core needle biopsy: Right breast invasive carcinoma, non-specific type, histological grade III. Immunohistochemistry: ER(-),PR(-), Her-2(2+),Ki-67(70%+).FISH testing indicates no HER-2 amplification. Based on imaging studies and needle biopsy pathology, a preliminary diagnosis of triple-negative breast cancer in the right breast has been made. Colonoscopy: Performed due to altered bowel habits and rectal bleeding. Findings suggest colorectal cancer and colonic polyps. Pathology indicates that the majority of the resected sigmoid colon specimen shows necrosis, with focal morphology consistent with moderately differentiated adenocarcinoma.

Following a multidisciplinary team (MDT) discussion involving breast surgery, gastrointestinal surgery, breast medicine, breast radiation oncology, radiology, and anesthesiology, an in-depth analysis was conducted regarding the treatment sequence and plan for the two primary cancers. First, concerning the treatment sequence, the team decided to adopt a “breast cancer first, sequential management of rectal cancer” strategy. The rationale is as follows: ①Tumor Biology: The patient has triple-negative breast cancer (TNBC), a subtype characterized by high proliferative activity (Ki-67: 70%+) and a high risk of early recurrence, necessitating prompt intervention. ② Disease burden and urgency: Although rectal cancer presented with rectal bleeding, CT evaluation showed no signs of acute obstruction or perforation, allowing for short-term deferral. In contrast, the breast tumor was confirmed malignant with suspected lymph node metastasis. Prioritizing its treatment avoids delays in systemic breast cancer therapy caused by subsequent major colorectal surgery and recovery (typically requiring 2–4 weeks); ③Patient Tolerance: Simultaneous radical resection at two sites would impose substantial trauma, significantly increasing surgical risk, prolonging recovery time, and potentially delaying timely chemotherapy. A sequential surgical approach is safer and more feasible. Secondly, regarding surgical modality selection: For breast cancer, given the tumor’s maximum diameter of approximately 2 cm, its location in the outer quadrant, and the patient’s desire for breast conservation, clear indications exist for breast-conserving surgery. This approach maximizes breast contour preservation and quality of life while ensuring oncological safety (negative margins). Concurrently, axillary lymph node dissection was performed to achieve local regional control due to positive sentinel lymph node biopsy. For rectal cancer, preoperative CT indicated the tumor was located in the upper rectum without sphincter involvement, meeting criteria for sphincter-preserving surgery. Therefore, laparoscopic radical resection of rectal cancer (Dixon procedure) was selected. Laparoscopic minimally invasive surgery offers advantages of reduced postoperative pain and faster recovery, facilitating the patient’s prompt transition to adjuvant therapy following two surgeries.

On April 29, 2025, underwent “right breast-conserving surgery for breast cancer + sentinel lymph node biopsy + axillary lymph node dissection.” Postoperative pathology: Right breast invasive carcinoma (2.4 × 1.7 × 1.4 cm), with cancer metastasis detected in one of three sentinel axillary lymph nodes. Immunohistochemistry: ER (negative), PR (negative), Her-2 (2+), Ki-67 (60%+).FISH testing confirmed no HER-2 amplification. Genetic testing: No mutations detected in the BRCA1 gene or BRCA2 gene. Postoperative diagnosis: Triple-negative breast cancer in the right breast (T2N1M0, Stage IIb).

**Figure 4:**
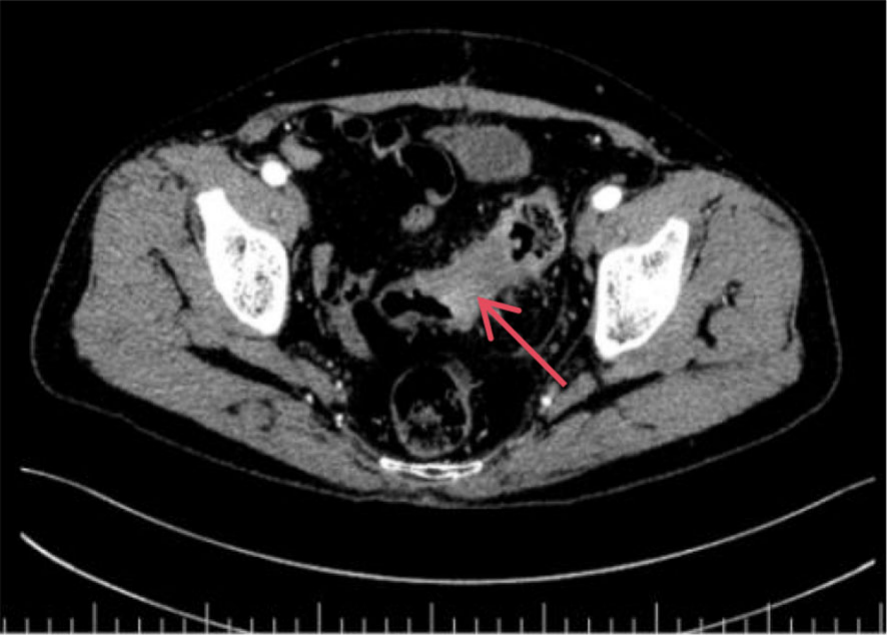
Enhanced CT of the lower abdomen and pelvis: The sigmoid colon wall appears rigid with marked heterogeneous thickening. The lumen shows irregular narrowing. The inner margin of the lesion is irregularly indented, while the outer margin is rough. After contrast enhancement, the affected bowel wall demonstrates markedly heterogeneous enhancement. The peritoneal fat space appears hazy. Multiple soft tissue density nodular shadows are visible around the bowel, with the largest measuring approximately 1.1 × 1.0 cm. (The red arrow in the image points to the affected area.).

**Figure 5:**
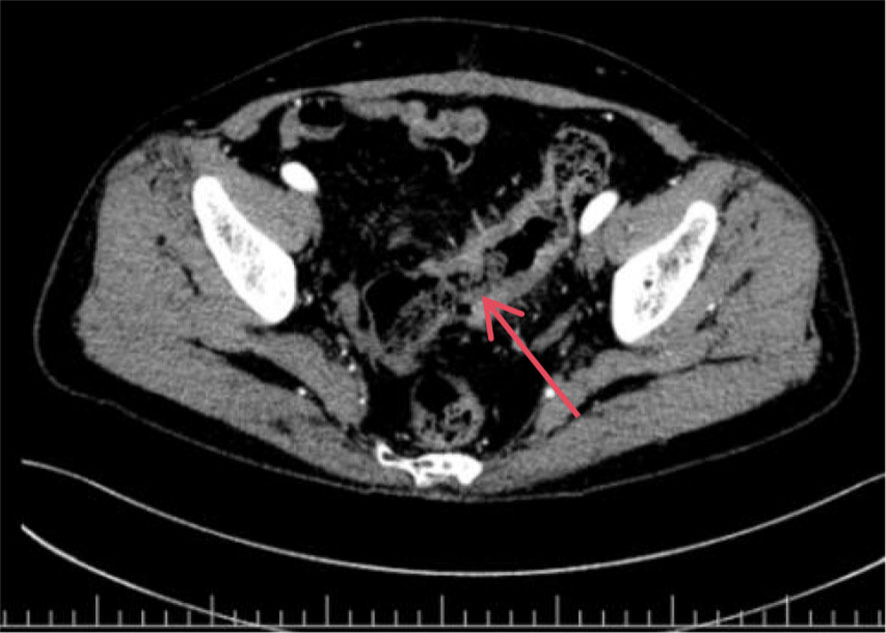
Enhanced CT of the lower abdomen and pelvis: The sigmoid colon wall appears rigid with marked heterogeneous thickening. The lumen shows irregular narrowing. The inner margin of the lesion is irregularly indented, while the outer margin is rough. After contrast enhancement, the affected bowel wall demonstrates markedly heterogeneous enhancement. The peritoneal fat space appears hazy. Multiple soft tissue density nodular shadows are visible around the bowel, with the largest measuring approximately 1.1 × 1.0 cm.(The red arrow in the image points to the affected area.).

After a 2-week rest period, the patient underwent contrast-enhanced CT, which revealed: Uneven thickening of the sigmoid colon wall, suggestive of colon cancer (**Figures 4**, **5**). Based on imaging studies and clinical findings, the patient underwent “laparoscopic radical resection of the rectum” on May 13, 2025.Postoperative pathology: Moderately differentiated adenocarcinoma of the rectal ulcer type (5×5×1.7 cm), invading the subserosal layer with visible vascular and lymphatic invasion; no lymph node metastasis detected (0/18).Postoperative diagnosis: Rectal adenocarcinoma (pT3N0M0, Stage IIa).The gene test indicates microsatellite stability (MSS).

## 3 Treatment

Following two surgical procedures, the MDT team reconvened to discuss adjuvant chemotherapy regimens. The rationale for administering systemic therapy “postoperatively” rather than “preoperatively” was as follows: For this patient’s breast cancer (Stage IIb) and rectal cancer (Stage IIa), there were no clear strong indications for neoadjuvant therapy prior to surgery (e.g., massive breast tumor requiring downstaging for breast conservation, or rectal cancer at T4 or N2 stage necessitating downstaging). The postoperative adjuvant treatment approach represents a guideline-compliant standard choice. Its advantages include enabling precise staging based on final pathology results to guide treatment planning, while avoiding potential surgery delays or cumulative toxicity associated with preoperative chemotherapy.

The 2025 CSCO Guidelines for the Diagnosis and Treatment of Triple-Negative Breast Cancer recommend AC-T as a Level I recommendation and TP regimen as a Level II recommendation.This patient underwent sphincter-preserving surgery for rectal cancer (T3N0M0). Pathology indicated vascular invasion, classifying the patient as a high-risk Stage II case. High-risk factors for Stage II patients include: T4, poor histological differentiation, vascular invasion, nerve infiltration, preoperative intestinal obstruction or tumor perforation, positive or indeterminate margins, insufficient margin clearance, and fewer than 12 lymph nodes examined). For high-risk stage II postoperative patients, the Level I recommendation in the latest 2025 CSCO Guidelines for the Diagnosis and Treatment of Rectal Cancer is combination chemotherapy (CAPEOX: oxaliplatin + capecitabine).Extensive research has demonstrated that oxaliplatin and carboplatin share a core mechanism of action: the formation of DNA adducts, which inhibits DNA replication and induces apoptosis in cancer cells (23). Both require hydrolysis within the body to remove their respective carrier ligands—cyclobutanedicarboxylic acid for carboplatin and oxalic acid and the DACH ring for oxaliplatin—releasing the biologically active hydrated platinum species (24). This active platinum center is key to attacking DNA. The resulting Pt-DNA adduct physically distorts the DNA double helix structure, acting like a “roadblock” that prevents DNA replication and transcription from proceeding. If this lethal DNA damage cannot be effectively repaired by the cell, it will ultimately activate the programmed cell death (apoptosis) pathway (23). Their clinical differences—such as drug resistance, toxicity, and indications—primarily stem from variations in pharmacokinetic properties and DNA adduct structures/repair mechanisms caused by their distinct inactive ligands.

Based on the above background, the MDT team discussed and ultimately selected the TP regimen (albumin-bound paclitaxel + carboplatin) due to the dual efficacy of platinum-based drugs in treating both breast cancer and colorectal cancer. The proposed treatment plan consists of 6 cycles of TP regimen followed by radiotherapy and capecitabine. This decision was based on the following considerations: ①Treatment Spectrum Coverage: The TP regimen falls within the recommended or commonly used treatment categories for both TNBC and colorectal cancer. As a platinum-based agent, carboplatin exhibits DNA cross-linking activity effective against both tumor types.② Synergistic effect: Taxanes and platinum-based drugs demonstrate synergistic antitumor effects in TNBC, particularly in tumors exhibiting BRCAness phenotypes. ③Efficiency-tolerability balance: Selecting a single sequential chemotherapy regimen for both primary cancers improves treatment efficiency compared to two distinct regimens for each cancer, reduces uncertainty from regimen switching, and may lower cumulative toxicity (e.g., oxaliplatin-specific neurotoxicity). Although CAPEOX is the standard regimen for stage II high-risk colorectal cancer, the TP regimen is prioritized for controlling highly aggressive TNBC while also addressing rectal cancer.

The patient initiated adjuvant chemotherapy with the TP regimen (albumin-bound paclitaxel + carboplatin) on June 3, 2025. Grade IV bone marrow suppression with fever developed after Cycle 1, resolving with short-acting G-CSF (Neupogen^®^), anti-infective therapy, and supportive care. Starting from Cycle 2, prophylactic long-acting G-CSF (Tolevol^®^) was intensified, improving tolerability and enabling timely completion of treatment. To date, the patient has completed 6 cycles of chemotherapy with stable disease. During the three-month follow-up period, imaging studies revealed no evidence of disease recurrence or metastasis, indicating that the patient’s condition remains stable.

## 4 Discussion

This case report describes a 52-year-old female patient diagnosed with dual primary ectopic malignancies: triple-negative breast cancer (TNBC) in the right breast and rectal adenocarcinoma. The diagnostic and treatment process involves multidisciplinary collaboration (MDT), decision-making on therapeutic strategies, comprehensive tumor management throughout the entire disease cycle, and management of chemotherapy-related adverse reactions, providing valuable clinical insights.

### 4.1 Discussion on the etiology and genetic susceptibility of dual-origin metastatic cancer

Breast cancer and colorectal cancer are both highly prevalent malignant tumors worldwide, but cases of multiple primary ectopic occurrence are relatively rare. Its occurrence may be associated with shared genetic background, environmental factors, or treatment-related factors. Although this patient has no clear family history of cancer, the pattern of dual cancer occurrence suggests we should remain vigilant for the possibility of hereditary cancer syndromes (25). The development of breast cancer is a series of events driven by the combined effects of genetic and environmental factors, propelling normal cells through a multistage transformation process involving hyperplasia, precancerous lesions, and carcinoma in situ (26). Genetic susceptibility is the primary and most significant factor. Genetic susceptibility to breast cancer arises from germline mutations in one allele of a moderately to highly penetrant susceptibility gene (such as BRCA1/2, CHEK2, PALB2, and TP53) (27–29). According to statistics, 7%–20% of TNBC patients carry BRCA1 or BRCA2 hereditary mutations, with approximately 80% of BRCA1 mutations detected in TNBC (30). For example, germline mutations in the BRCA1/2 genes, which are strongly associated with breast cancer, have also been found to be linked to a slightly increased risk of colorectal cancer. However, the patient’s genetic testing indicated no detectable mutations in the BRCA1 or BRCA2 genes, thus ruling out lesions caused by BRCA1/2 germline mutations. Additionally, Lynch Syndrome, the most common hereditary colorectal cancer syndrome caused by mismatch repair (MMR) gene mutations, carries the highest risk of endometrial cancer (EC) among female carriers. However, whether it increases the risk of breast cancer remains controversial (31). The postoperative pathology of this patient with colorectal cancer indicated pMMR (microsatellite stability), reducing the likelihood of Lynch syndrome but not completely ruling out other genetic predisposing factors (such as PALB2, CHEK2, etc.).Therefore, comprehensive genetic counseling is crucial for patients with multiple-source ectopic carcinoma. This not only aids in explaining the etiology but also provides significant guidance for risk assessment and screening strategy development among their first-degree relatives (32).

In cases of dual-primary malignancies, comprehensive multigene panel testing for hereditary cancer syndromes is strongly recommended to delineate the complete spectrum of genetic susceptibility and guide personalized surveillance strategies. Beyond the well-established BRCA1/2 and Lynch syndrome genes, large panels can identify pathogenic variants in other moderate-to-high penetrance genes, such as PALB2 and CHEK2, which are implicated in both breast and colorectal cancer risks (33). The identification of a mutation in these genes directly informs tailored, syndrome-specific surveillance protocols. For instance, carriers of a PALB2 pathogenic variant require enhanced breast screening with annual MRI starting at a young age. Furthermore, emerging evidence from studies on cancers of unknown primary (CUP) demonstrates that broader genomic analyses, including whole-genome and transcriptome sequencing (WGTS), can uncover additional diagnostically and therapeutically relevant alterations beyond what is detectable by limited panels, thereby refining risk management approaches. This genetic risk stratification, coordinated through a multidisciplinary team (MDT), ensures the implementation of proactive cancer prevention, early detection, and risk-reducing interventions for high-risk patients and their families.

### 4.2 Treatment strategy selection and the core role of multidisciplinary teamwork

For multiple primary malignant tumors, determining the sequence of treatment represents the primary challenge in clinical decision-making. The MDT team in this case decided to prioritize breast cancer surgery based on the following considerations: ①The breast cancer is a triple-negative subtype, characterized by high aggressiveness and a significant risk of early recurrence, necessitating prompt intervention; ②The colorectal cancer, detected due to “blood in stool,” has persisted for one year but imaging studies show no signs of advanced disease or obstruction, allowing for short-term deferral of treatment; ③Addressing the breast cancer first avoids potential delays in breast cancer treatment (particularly chemotherapy) caused by subsequent colorectal cancer surgery and recovery. This embodies the core principle of the MDT model: “patient-centered care and the development of personalized optimal solutions.” Following breast cancer surgery, a radical resection for rectal cancer was promptly performed, followed by combined chemotherapy using the TP regimen (albumin-bound paclitaxel plus carboplatin). This regimen falls within the recommended treatment categories for both triple-negative breast cancer (TNBC) and rectal cancer, maximizing therapeutic efficacy (32).

### 4.3 Prospects for alternative treatment strategies

Although this patient achieved favorable outcomes with standard treatment based on surgery, chemotherapy, and radiotherapy, the field of tumor therapy is advancing rapidly, with multiple emerging strategies offering potential options for similar patients in the future. In gene therapy, genome editing technologies such as CRISPR/Cas9 have been explored to correct oncogenic gene mutations (e.g., p53) or enhance anti-tumor immunity [21]. MicroRNAs (miRNAs) and long non-coding RNAs (lncRNAs), as key regulators of gene expression, play significant roles in the development, metastasis, and drug resistance of TNBC and colorectal cancer. Research into their use as prognostic biomarkers and therapeutic targets is gaining momentum [22]. For example, restoring the miR-200c family, which is downregulated in TNBC, or targeting the oncogenic lncRNA H19 may offer novel pathways to inhibit tumor progression. Furthermore, stem cell-based therapies, particularly those targeting cancer stem cells (CSCs), aim to eradicate the root causes of tumor recurrence and metastasis, representing a current hotspot in translational research [23]. Although most of these cutting-edge therapies remain in preclinical or early clinical trial phases, still distant from routine clinical application, they signify the future direction of tumor treatment—shifting from “standardized” approaches toward ‘personalized’ and “root-cause” therapies.

### 4.4 The challenges of chemotherapy toxicity and the importance of supportive care

Following the first cycle of TP chemotherapy regimen, the patient developed Grade IV bone marrow suppression with fever (febrile neutropenia, FN), one of the most severe dose-limiting toxicities of chemotherapy. This incident highlights that patients with dual cancers undergoing intensive chemotherapy may face greater challenges in terms of physical fitness and bone marrow reserve. The clinical team promptly implemented standardized management protocols, including granulocyte colony-stimulating factor (G-CSF) administration to boost white blood cell counts, prophylactic isolation, and empirical antimicrobial therapy. This enabled the patient to recover and successfully complete subsequent treatment. This experience reminds us that: ①For similar high-risk patients, prophylactic use of G-CSF after the first cycle of chemotherapy may be considered to reduce the risk of FN (34); ②Comprehensive education on post-chemotherapy self-management must be provided to patients and their families; ③Blood counts should be closely monitored during subsequent treatment cycles, with readiness for prompt and robust supportive care to ensure treatment safety and adherence, thereby optimizing the comprehensive tumor management model.

## 5 Conclusion

The successful diagnosis and treatment of this case of breast cancer with concurrent rectal cancer as multiple primary malignant tumors was highly dependent on the scientific and rational sequential treatment strategy formulated by the multidisciplinary team (MDT) following a comprehensive assessment of the patient’s condition. The diagnostic and treatment process underscores that for patients with multifocal ectopic carcinoma, routine assessment of genetic susceptibility should be conducted. Utilizing a multidisciplinary team (MDT) platform, treatment sequencing and regimens should be individualized based on the tumor’s biological behavior, urgency, and prognosis. At the same time, it is crucial to pay close attention to the toxic side effects associated with high-intensity combination therapies. Strengthening preventive and therapeutic supportive measures while enhancing awareness of comprehensive tumor management throughout the entire treatment cycle will ultimately ensure both effective tumor control and the safety and quality of life for patients. The patient is currently in remission, and long-term prognosis requires ongoing follow-up observation. This case underscores the importance of genetic risk assessment for patients with dual primary ectopic cancers, developing individualized treatment plans through multidisciplinary team collaboration, and enhancing supportive care. It provides valuable reference for the clinical management of similar patients.

## Data Availability

The original contributions presented in the study are included in the article/supplementary material. Further inquiries can be directed to the corresponding author.
